# A Three-Dimensional, Magnetic and Electroactive Nanoprobe for Amperometric Determination of Tumor Biomarkers

**DOI:** 10.3390/ijms12010362

**Published:** 2011-01-14

**Authors:** Linghua Meng, Ning Gan, Tianhua Li, Yuting Cao, Futao Hu, Lei Zheng

**Affiliations:** 1 The State Key Laboratory Base of Novel Functional Materials and Preparation Science, Faculty of Material Science and Chemical Engineering, Ningbo University, Ningbo, Zhejiang 315211, China; E-Mails: meng_ting_2008@163.com (L.M.); litianhua@nbu.edu.cn (T.L.); caoyuting@nbu.edu.cn (Y.C.); hufutao@nbu.edu.cn (F.H.); 2 Clinical Laboratory Center, Nanfang Hospital, Southern Medical University, Guangzhou, Guangdong 510515, China

**Keywords:** alpha-fetoprotein, nanoprobes, screen-printed carbon electrode, electrochemical immunosensor

## Abstract

A novel electrochemical immunosensor for tumor biomarker detection based on three-dimensional, magnetic and electroactive nanoprobes was developed in this study. To fabricate the nanoprobes, negatively charged Fe_3_O_4_ nanoparticles (Fe_3_O_4_ NPs) and gold nanoparticles (Au NPs) were first loaded on the surface of multiple wall carbon nanotubes (MCNTs) which were functioned with redox-active hemin and cationic polyelectrolyte poly(dimethyldiallylammonium chloride) (PDDA). Using alpha fetoprotein (AFP) as a model analyte, AFP antibody (anti-AFP) was absorbed on the surface of Au NPs, bovine serum albumin (BSA) was then used to block sites against non-specific binding, and finally formed anti-AFP/Au NPs/Fe_3_O_4_/hemin/MCNTs named anti-AFP nanoprobes. When the target antigen AFP was present, it interacted with anti-AFP and formed an antigen-antibody complex on the nanoprobe interface. This resulted in a decreased electrochemical signal of hemin for quantitative determination of AFP when immobilized onto the screen-printed working electrode (SPCE). The results showed that the nanoprobe-based electrochemical immunosensor was sensitive to AFP detection at a concentration of 0.1 to 200 ng·mL^−1^ with a detection limit of 0.04 ng·mL^−1^, it also demonstrated good selectivity against other interferential substances. The electroactive nanoprobes can be massively prepared, easily immobilized on the SPCE for target detection and rapidly renewed with a magnet. The proposed immunosensor is fast, simple, sensitive, stable, magnet-controlled, nontoxic, label-free and reproducible.

## 1. Introduction

In recent years, protein detection in biological samples has received considerable attention in disease diagnosis, food safety control, environmental monitoring and many other fields. Especially, the detection of low-concentration tumor biomarkers is very important for early diagnosis of diseases [1]. Immunoassay, as a promising approach for selective and sensitive analysis, has recently gained increasing attention in the quantitative detection of tumor markers and screening of cancers [2]. Among the currently variable immunoassay protocols, electrochemical immunosensors, based on specific antibody-antigen interaction, is one of the most powerful tools for sensitive detection of tumor biomarkers due to the features of fast analysis, simple preparation, low detection limit and precise measurement [3,4]. More recently, various electrochemical immunosensors have been developed; however, many of them usually need complicated enzyme-labeling procedures, or some need the addition of mediator to the detection buffer, or require a relatively long assay time [5–7]. Therefore, developing novel, fast, label-free, simple, nontoxic and reproducible assay systems is a challenging topic in the fabrication of a sensitive electrochemical immunoassay.

During the fabrication of electrochemical immunosensors, biomolecular immobilization is vital in successful construction of an immunosensor [8]. Ideally, antibody immobilization methods in immunosensors should allow for high loading, retention of long-term biological activity, simplicity and easy control [9,10]. To meet these challenges, various types of nanomaterials have been investigated as substrates for the immobilization of antibodies. For example, gold nanoparticles (Au NPs) have been widely used due to their desirable characteristics: large specific surface area, strong adsorption ability, and good conductivity [11,12]. Carbon nanotubes (CNTs) have also been extensively used as an immobilized substrate owing to their unique properties of high chemical stability, good electrical conductivity, high surface-to-volume ratio and strong adsorptive ability. Besides, the three dimensional CNT nanostructures can largely enhance the immobilized amount of protein [13–15]. Recently, magnetic nanoparticles have also gained increasing interest and have been widely applied in immunoassays [16,17] due to their biocompatibility, superparamagnetism and good electron conductivity [18], which can simplify the process of protein immobilization and separation [19]. The magnetic nanoprobes strategy developed recently has proven to be a highly sensitive technique for detecting human tumor cells, and is especially well suited to separate and in the meantime detect low-concentrations of proteins [20,21].

Another important issue in electrochemical immunosensors is converting the specific antibody-antigen interaction to a detectable electrochemical signal so as to quantitatively determine the concentration of target antigen. Using the direct electrochemistry of immobilized electron mediator for signal converting can bring about a simple and efficient sensor design. Recently, Du *et al.* developed an electrochemical biosensor for protein detection using layer-to-layer self-assembly to co-immobilize target-specific aptamer and redox-active ferrocene-appended poly(ethyleneimine). The results demonstrated that the sensor design integrating recognition element and electron mediator could provide a label-free and highly sensitive detection platform for proteins [22].

**Scheme 1:**
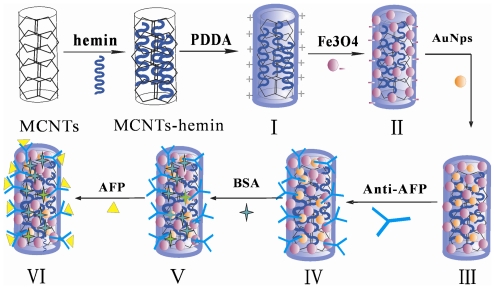
The preparation procedure for AFP nanoprobes. (**I**): MCNTs-hemin-PDDA; (**II**): Fe_3_O_4_/hemin/MCNTs nanocomposites; (**III**): Au NPs/Fe_3_O_4_/hemin/MCNTs; (**IV**): anti-AFP/Au NPs/Fe_3_O_4_/hemin/MCNTs; (**V**): BSA anti-AFP/Au NPs/Fe_3_O_4_/hemin/MCNTs; (**VI**): AFP/anti-AFP/Au NPs/Fe_3_O_4_/hemin/MCNTs.

In this work, with a view to overcome the challenge for developing a label-free, simple, fast, nontoxic and reproducible assay system, a novel electrochemical immunosensor for tumor biomarkers has been demonstrated by co-immobilizing target-specific aptamer and electron mediator on magnetic nanoprobes. The nanoprobes were fabricated by incorporating antibody into a new nanocomposite fabricated by loading Au NPs and Fe_3_O_4_ NPs on the surface of multiple wall carbon nanotubes (MCNTs) functioned with redox-active hemin and cationic polyelectrolyte poly (dimethyldiallylammonium chloride) (PDDA). Hemin (iron protoporphyrin IX) is the active center of many hemin-containing redox proteins, it exhibits direct electron transfer independently of the orientation on the electrode surface due to its small size and has been used as an electrochemical indicator in sensor design [23,24]. Furthermore, hemin contains a porphyrin ring, which may be immobilized at the surface of MCNTs through non-covalent functionalization by π-π interaction [25]. PDDA is a water-soluble, quaternary ammonium, cationic polyelectrolyte that usually acts as a positively charged colloid when dissolved in aqueous solutions [26,27] and can be easily coated on the negatively charged surface of the MCNTs by electrostatic interactions. As shown in Scheme 1, the negatively charged Fe_3_O_4_ NPs and Au NPs were sequentially assembled onto the surface of MCNTs which were functioned with hemin and PDDA to prepare Au NPs/Fe_3_O_4_/hemin/MCNTs nanocomposite through electrostatic interaction. Using alpha fetoprotein (AFP) as a model antigen, AFP antibody (anti-AFP) was absorbed on the surface of Au NPs, bovine serum albumin (BSA) was then used to block sites against non-specific binding and finally formed anti-AFP/Au NPs/Fe_3_O_4_/hemin/MCNTs named anti-AFP nanoprobes. When the target antigen AFP is present, it interacts with anti-AFP and forms an antigen-antibody complex on the nanoprobe interface, which affects electron transfer and results in a decreased electrochemical signal of hemin for quantitative determination of AFP when immoblizing them onto the screen-printed working electrode (SPCE). The method combined the following advantages: (1) Hemin and anti-AFP co-immobilized on magnetic nanoprobes and the detection of AFP was realized through a one-step immunoassay format that could bring in a simple, label-free and sensitive sensor design. (2) The Au NPs/Fe_3_O_4_/hemin/MCNTs hybrids, used as solid support for capturing anti-AFP, not only facilitated magnet-mediated separation and fast detection, but also retained the biological activity of protein. (3) The nanoprobes can be prepared in advance, are easily immobilized on the SPCE for target detection and rapidly renewed with a magnet after each determination. The proposed immunosensor is fast, simple, sensitive, stable, magnet-controlled, nontoxic, label-free and reproducible.

## 2. Results and Discussion

### 2.1. Characterization of Different Nanoparticle Complexes

**Figure 1:**
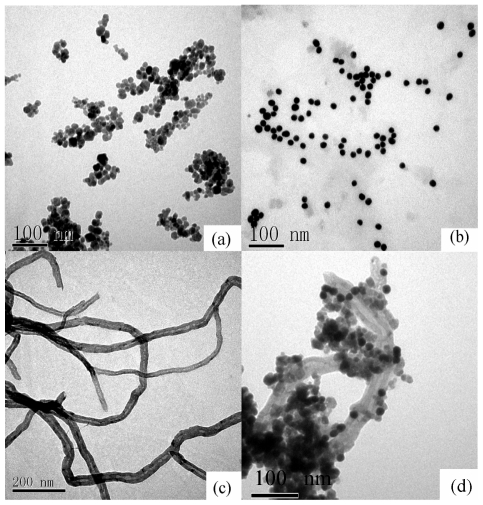
TEM images of (**a**) Fe_3_O_4_ NPs, (**b**) AuNPs, (**c**) MCNTs and (**d**) Au NPs/Fe_3_O_4_/hemin/MCNTs nanocomposite.

The Fe_3_O_4_ NPs, Au NPs, MCNTs and Au NPs/Fe_3_O_4_/hemin/MCNT nanocomposites were characterized by transmission electron microscopy (TEM). As shown in Figure 1, nearly each Fe_3_O_4_ nanoparticle (Figure 1a) was neatly separated from its neighbor due to the surfactants absorbed on the surface, and the average diameter of Fe_3_O_4_ NPs obtained from TEM images was about 20 nm. Figure 1b shows that the Au NPs were independent of each other, the purified MCNTs showed smooth and uniform surface morphology (Figure 1c). After MCNTs were functioned with hemin and PDDA, the negatively charged Fe_3_O_4_ NPs and Au NPs were easily assembled on the surface of MCNTs through electrostatic interactions. Figure 1d displays that numerous Fe_3_O_4_ NPs and some independent Au NPs were successfully absorbed onto the surface of the MCNTs-hemin. X-ray fluorescence spectrometry (determination of elements in the scope of ^9^F~^92^U) was further used to confirm if the Au NPs were assembled on the Fe_3_O_4_/hemin/MCNTs nanocomposite. The results showed the characteristic peaks of Fe (*k*α−6.4 keV) and Au (*L*A−9.7 keV) (data not shown), indicating the successful immobilization of Au NPs on the surface of Fe_3_O_4_/hemin/MCNTs nanocomposites.

**Figure 2:**
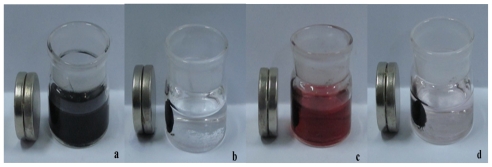
The different synthesis stage of the nanoprobes under external magnetic field. (**a**) MCNTs-hemin solution; (**b**) Fe_3_O_4_/hemin/MCNTs hybrids; (**c**) Fe_3_O_4_/hemin/MCNTs mixed with Au NPs initially; (**d**) Au NPs/Fe_3_O_4_/hemin/MCNTs composites.

Figure 2 illustrates the different synthesis stages of the nanoprobes in the presence of an external magnetic field. The MCNTs-hemin solution showed a stable state of homogeneous dispersity, and could not be aggregated by a magnet (Figure 2a). When the solution was mixed with negatively charged Fe_3_O_4_ NPs and stirred for 30 min, the formed suspension could be easily separated from the solution (Figure 2b), implying the successful formation of magnetic Fe_3_O_4_/hemin/MCNTs hybrids. The assembly of Au NPs was also confirmed. Figure 1c showed that only the Fe_3_O_4_/hemin/MCNTs hybrids were aggregated in the initial stage of mixing Au NPs and Fe_3_O_4_/hemin/MCNTs suspensions. After being sufficiently stirred, the characteristic rose-red color of the Au NPs solution diminished (Figure 2d), which indicated the Au NPs were absorbed onto the Fe_3_O_4_/hemin/MCNTs composites and separated from the solution.

### 2.2. Cyclic Voltammetry (CV) Measurements of the Different Nanoparticle Modified Electrodes

**Figure 3:**
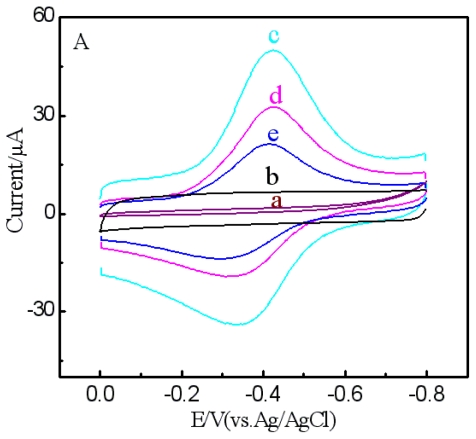
Cyclic voltammogram of different composite modified SPCEs. (**a**) bare SPCE; (**b**) SPCE|MCNTs-Fe_3_O_4_; (**c**) SPCE|Fe_3_O_4_/hemin/MCNTs; (**d**) SPCE|BSA/anti-AFP/Au NPs/Fe_3_O_4_/hemin/MCNTs; (**e**) SPCE|AFP/anti-AFP/Au NPs/Fe_3_O_4_/hemin/MCNTs in 0.1 mol/L PBS (pH = 6.5) at a scan rate of 100 mV/s.

The electrochemical behaviors of different nanoparticle modified electrodes in 0.1 M PBS (pH = 6.5) were studied by CV and the results are shown in Figure 3. No redox peak was found at the bare electrode (Figure 3a). After being modified with Fe_3_O_4_-MCNTs, the bared electrode showed increased background current and remained redox-silent (Figure 3b), indicating that the modification of MCNTs and Fe_3_O_4_ significantly enhanced the electrical conductivity of the electrode. However, when Fe_3_O_4_/hemin/MCNTs were deposited on the SPCE, a couple of redox peaks were observed, suggesting that direct electron transfer of hemin was achieved (Figure 3c). After anti-AFP and BSA were absorbed on the Au NPs/Fe_3_O_4_/hemin/MCNTs hybrids to form the anti-AFP nanoprobes, the current response of hemin reduced (Figure 3d); this current decrease should be attributed to the immobilized anti-AFP and BSA hindering the electron transfer from hemin to the SPCE. After the anti-AFP nanoprobes were incubated with AFP solution, a further current decrease was observed (Figure 3e), indicating the formation of antigen-antibody complexes on the anti-AFP nanoprobes, which further inhibited the electrochemical communication between hemin and the SPCE.

### 2.3. Optimization of Experimental Conditions

#### 2.3.1. The Ratio of Fe_3_O_4_ Conjugated to MCNTs-hemin and Quantity of Fe_3_O_4_/hemin/MCNTs Nanocomposites on SPCE

**Figure 4:**
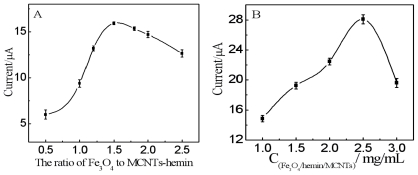
(**A**) Optimization of reaction ratio of Fe_3_O_4_ to MCNTs-hemin; (**B**) Effect of quantity of Fe_3_O_4_/hemin/MCNTs nanocomposites on the surface of SPCE.

The optimum ratio of Fe_3_O_4_ conjugated to MCNTs-hemin was studied by measuring the current signal of Fe_3_O_4_/hemin/MCNTs composites with different quantities of Fe_3_O_4_ on SPCE. As shown in Figure 4A, the current response gradually increased with the ratio of Fe_3_O_4_ to MCNTs-hemin from 0.5:1.0 to 1.5:1.0. At higher ratios over 1.5:1.0, the current response decreased, probably due to the aggregation of Fe_3_O_4_ NPs, which caused decrease of their conductivity. Based on the result, the optimum ratio of Fe_3_O_4_ to MCNTs-hemin was selected as 1.5:1.0. The effect of Fe_3_O_4_/hemin/MCNTs content in the suspension deposited on SPCE was examined from 1.0 to 3.0 mg·mL^−1^ (Figure 4B). The current response gradually increased with the increasing of Fe_3_O_4_/hemin/MCNTs content from 1.0 to 2.5 mg·mL^−1^. At higher concentrations over 2.5 mg·mL^−1^, the current response decreased. Thus, the optimum concentration of Fe_3_O_4_/hemin/MCNTs was determined as 2.5 mg·mL^−1^.

#### 2.3.2. The Maximum Quantity of Anti-AFP Immobilized on the Au NPs/Fe3O4/hemin/MCNTs Nanocomposite

**Figure 5:**
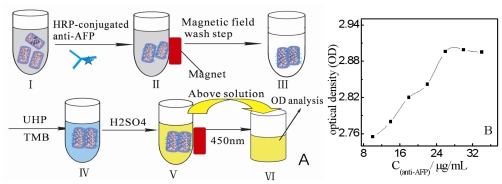
(**A**) Spectrophotometric analysis procedure of maximum quantity of anti-AFP immobilized on the 1 mg Au NPs/Fe_3_O_4_/hemin/MCNTs. (I): 2.0 mL pH-adjusted Au NPs/Fe_3_O_4_/hemin/MCNTs composites; (II): (I) reacted with different concentration of anti-AFP; (III): the formed anti-AFP nanoprobes were separated and washed three times using magnet and redispersed in pH 6.5 PBS; (IV): Addition to suspension of 50 μL UHP and TMB, the solution turned blue immediately; (V): After 5 min, the above reaction was blocked with 2 M H_2_SO_4_ and the solution turned yellows; (VI): the above solution was seperated from anti-AFP nanoprobes using a magnet and then transferred to the 96-well plastic plate for OD analysis at the wavelength of 450 nm. (**B**) the analysis result of maximum quantity of anti-AFP immobilized on the 1 mg Au NPs/Fe_3_O_4_/hemin/MCNTs.

In this experiment, we used HRP-anti-AFP to obtain the maximum quantity of AFP antibody could immobilize on the Au NPs/Fe_3_O_4_/Hemin/MCNTs nanocomposite. The binding quantity of AFP antibody was evaluated by measuring the optical density (OD) at 450 nm after the chromogenic reaction of HRP conjugated to anti-AFP catalyzed the oxidation of tetramethylbenzidine (TMB) by Urea Hydrogen Peroxide (UHP) (Figure 5A). There was nearly no negative influence of HRP on electrochemical response of hemin (date not shown). The analysis result is shown in Figure 5B, and it can be seen that the OD value increased with the increasing anti-AFP concentration and achieved a constant value at the anti-AFP concentration of 26 μg·mL^−1^. Thus, anti-AFP of 26 μg·mL^−1^ was chosen as the optimum concentration to be absorbed on 1 mg Au NPs/Fe_3_O_4_/hemin/MCNTs.

#### 2.3.3. Effect of Incubation Time and pH of the Working Buffer

**Figure 6:**
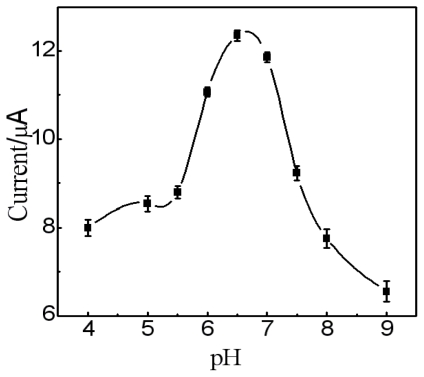
The dependence of amperometric response on the pH of the working buffer.

Incubation time for the antigen-antibody interaction was investigated. The anti-AFP nanoprobes were incubated with 20 ng·mL^−1^ AFP solution at 25 °C for different time periods and then electrochemical detection was carried out. The current response rapidly increased in the first 20 min and then leveled off (date not shown), indicating that an incubation time of 20 min was sufficient to achieve a complete antigen-antibody interaction. Therefore, an incubation time of 20 min was used for the detection of AFP. A strongly acidic or alkaline environment can destroy the protein microstructure and has an adverse effect on the protein activity. The effect of pH on the immunosensor responses was investigated by varying the pH of the working buffer from 4.0 to 9.0. As shown in Figure 6, the current response intensified as the pH increased and reached a maximum value at pH of 6.5. When the pH was over 6.5, the current response decreased. Accordingly, all subsequent electrochemical detections were carried out in pH 6.5 PBS.

### 2.4. Performance of the Immunosensor

**Figure 7:**
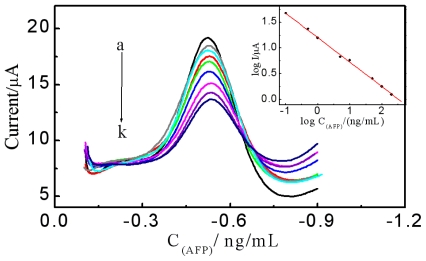
The differential pulse voltammetry (DPV) responses of the AFP nanoprobes in pH 6.5 PBS solution with different concentrations of AFP (0.0, 0.1, 0.5, 1.0, 5.0, 10, 50, 100, and 200 ng·mL^−1^ from a to k ) at 25 °C for 20 min. Inset: the relationship between log of DPV current signal towards log of different AFP concentrations.

Figure 7 shows differential pulse voltammetry (DPV) responses of the anti-AFP nanoprobes to different concentrations of AFP standards. As can be seen, under the optimal experimental conditions, the DPV signal decreased with the increase of AFP concentration, indicating that the formation of antigen-antibody immunocomplexes on the surface of the anti-AFP nanoprobes could effectively hinder the electron transfer from the SPCE working electrode to the immobilized redox-active hemin and the decreased DPV response could be used as a readout signal for quantitative analysis of AFP. As presented in the inset of Figure 7, the logarithm (log) of the DPV peak current was proportional to the log of AFP concentration from 0.1 to 200 ng·mL^−1^ with a correlation coefficient of 0.998. The detection limit was 0.04 ng·mL^−1^ at a signal-to-noise ratio of 3.

### 2.5. Selectivity of the Immunosensor

Possible interfering substances were also tested to evaluate the selectivity of the present immunosensor. The anti-AFP nanoprobes were incubated with 50 ng·mL^−1^ AFP or 50 ng·mL^−1^ AFP containing one of the following: carcinoembryonic antigen (CEA, 80 ng·mL^−1^), hepatitis B antigen (HBsAg, 20 ng·mL^−1^), human chorionic gonadotropin antigen (HCG, 20 ng·mL^−1^), BSA (2 μg·mL^−1^), ascrobic acid (AA, 2 μg·mL^−1^), dopamine (DA, 2 μg·mL^−1^) and L-lysine (2 μg·mL^−1^). The results demonstrated that the current responses of the two tested samples showed less than 5.9% difference, indicating that the developed immunosensor occupied a good selectivity for AFP detection against other interferential substances.

### 2.6. Application of the Immunosensor to Serum Samples

To demonstrate the practical application of the developed immunosensor in clinical analysis, four different concentrations of AFP standard solution were added to normal real human serum and examined by the proposed immunosensor and the enzyme-linked immunoassays (ELISA) method. The results are shown in Table 1, the relative errors between the two methods were from −6.0% to 5.7%, which indicated that the developed immunoassay methodology might be preliminarily applied for the determination of AFP in human serum for routine clinical diagnosis.

## 3. Experimental Section

### 3.1. Chemicals and Reagents

Gold chloride tetrahydrate, sodium citrate, ferric chloride (FeCl_3_·6H_2_O), ferrous chloride (FeCl_2_·4H_2_O), oleic acid (C_18_H_34_O_2_), ammonium hydroxide (25 wt% NH_3_ in water) were purchased from Sinophram chemical reagent Co. Ltd. PDDA (MW: 100,000–200,000 g·mol^−1^, in 20% aqueous solution), BSA and porcine hemin (99%) were purchased from Sigma Co. Ltd. MCNTs were purchased from Shenzhen Nanotech Port Co. Ltd. Horseradish peroxidase-conjugated murine monoclonal AFP antibody (0.5 mg·mL^−1^) and AFP-ELISA kits, including six AFP antigen standard solutions with various concentrations from 5 to 400 ng·mL^−1^, were purchased from ZhengZhou Biocell Biotechnology Co. Ltd. Phosphate buffer solution (PBS, 0.1 M, pH = 6.8) was used as supporting electrolyte. All stock and buffer solutions were prepared using double-deionized water (Milli-Q, Millipore Corporation).

### 3.2. Instruments and Measurements

CV and DPV measurements were carried out on a CHI 660B electrochemical analyzer (CH Instruments Co. USA). SPCE were purchased from eDAQ technology Corporation, Shanghai (a carbon electrode served as the working electrode, the auxiliary and reference electrodes were a carbon electrode and an Ag/AgCl electrode, respectively). A H-7650 transmission electron microscope (Hitachi, Japan) was employed to assess the morphology of the nanoparticles.

### 3.3. Synthesis of Oleic Acid-Coated Fe_3_O_4_ NPs

The oleic acid-coated Fe_3_O_4_ NPs were prepared by a facile one-pot method according to the literature [28]. Firstly, 4.3 g FeCl_2_·4H_2_O and 11.6 g FeCl_3_·6H_2_O were dissolved in 350 mL deionized water and heated to 80 °C under an N_2_ atmosphere with vigorous stirring. Then 20 mL of 25 wt% NH_4_OH was rapidly added into the above-prepared solution and vigorously stirred for 5 min. At last, 1 mL oleic acid was added into the suspension and allowed to react for 25 min to form the tar-like black magnetic gel precipitate. The obtained magnetic particles were sequentially washed with deionized water and ethanol to remove the excess oleic acid, and then dried in vacuum for use.

### 3.4. Synthesis of Au NPs

The gold nanoparticles were prepared according to the literature [29] by adding 2 mL of 1% (w/w) sodium citrate solution into 50 mL of 0.01% (w/w) HAuCl_4_ boiling solution. The solution was filtered with 0.22 μm microfiltration membrane and stored in a refrigerator in a dark-colored glass bottle before use. The mean size of the prepared Au NPs was about 16 nm, as confirmed by TEM.

### 3.5. Preparation of Anti-AFP and AFP Nanoprobes

MCNTs were purified by ultrasonication in 0.5 M HCl for 4 h. The products were washed with water and dried at 50 °C overnight. The purified MCNTs were first functioned with hemin by mixing 2.5 mg·mL^−1^ hemin and 1 mg·mL^−1^ MCNTs for 2 h. Then, 5.0 mg hemin-MCNTs were dispersed into 30 mL 1 wt% PDDA solution containing 0.5 M NaCl and stirred for 3 h, residual PDDA was removed by centrifugation. Finally, the PDDA coated hemin-MCNTs were mixed with negatively charged Fe_3_O_4_ NPs and the obtained magnetic Fe_3_O_4_/hemin/MCNTs nanocomposites were separated with a magnet. Above-prepared Fe_3_O_4_/hemin/MCNTs nanocomposites were dispersed in 9.0 mL of colloidal Au NPs and stirred for 30 min. The obtained Au NPs/Fe_3_O_4_/hemin/MCNTs were washed with water and dried in a vacuum oven. Then, 100 μL of 0.5 mg·mL^−1^ anti-AFP was added to 2.0 mL pH-adjusted Au NPs/Fe_3_O_4_/hemin/MCNTs composites. The mixture was treated by shaking table at 4 °C for 12 h, and then separated by magnet, and washed with pH 6.5 PBS three times. Following that, the anti-AFP/Au NPs/Fe_3_O_4_/hemin/MCNTs were treated with 3% BSA at 35 °C for 1 h to form anti-AFP nanoprobes. The synthesized anti-AFP nanoprobes were redispersed in PBS and stored at 4 °C when not in use.

The above anti-AFP nanoprobes were incubated with a series of AFP standard solutions in polyethylene tubes at 25 °C for 20 min to form magnetic and electroactive AFP/anti-AFP/Au NPs/Fe_3_O_4_/hemin/MCNTs (AFP nanoprobes), which were separated by magnetic decantation and washed with PBS three times.

### 3.6. The Detection Process of AFP on SPCE

**Scheme 2:**
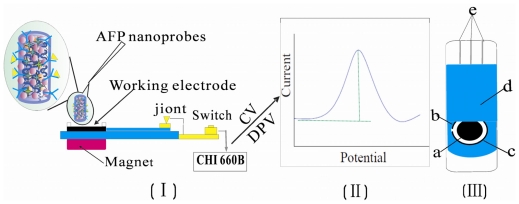
The detection process for different concentrations of AFP on SPCE. (**I**): different concentrations of AFP nanoprobes from 0 to 200 ng·mL^−1^ were dropped on the working electrode of SPCE; (**II**): the current signal recorded by DPV; (**III**): the planar graph of the three-electrode SPCE system. (a) Work electrode; (b) Ag/AgCl reference electrode; (c) Carbon counter electrode; (d) Insulator; (e) Joint.

A series of 6.5 μL AFP nanoprobes with different concentrations of AFP were dropped on the well-rinsed working electrode of SPCE with the aid of a magnet and 15 μL PBS was added as supporting electrolyte. The electrode was washed by ethanol and water three times prior to the next concentration of AFP determination, and the current signal was recorded by DPV. A nitrogen atmosphere was always maintained in the experiments. The detection process is shown in Scheme 2.

## 4. Conclusions

In this paper, we have fabricated a three-dimensional, magnetic and electroactive anti-AFP nanoprobe and applied it to detect AFP sensitively and selectively. Here, the magnetic and electroactive anti-AFP nanoprobes played multiple roles. Firstly, the Au NPs/Fe_3_O_4_/hemin/MCNTs hybrids were used as a solid support for capturing the sensor recognition element of anti-AFP, which facilitated magnet-mediated separation and fast detection. Secondly, the good electrical conductivity and biocompatibility of Au NPs, MCNTs and Fe_3_O_4_ NPs not only increased the immunoassay sensitivity but also retained the biological activity of AFP proteins. Thirdly, the one-step immunoassay format and the use of the direct electrochemical signal of immobilized hemin as sensor response offered a simple and label-free immunosensor platform for biomarker detecting. Furthermore, the nanoprobes can be largely prepared in advance, easily immobilized on the SPCE for target detection and rapidly renewed with a magnet after each determination. In addition, the reliability of the developed immunosensor has been proved by the satisfactory results of the usage to determine AFP in serum samples. Although this work only reported the detection of AFP *in vitro*, it is likely that the method will ultimately apply *in vivo* and for online detection. Therefore, the study provides a suitable method for the screen determination of low concentration of tumor markers in human serums and can be readily extended for determination of other clinically or environmentally interested biospecies.

## Figures and Tables

**Table 1:** Comparison of serum levels by using two methods: the proposed immunosensor and the enzyme-linked immunoassays (ELISA) method.

Added AFP Value (ng/mL)	5	10	20	50
**ELISA** (**ng/mL**)	5.18	10.36	21.33	49.75
**The Present Immunosensor** (**ng/mL**)	5.32	9.74	20.52	52.59
**Relative Deviations** (**%**)	2.7	−6.0	−3.8	5.7
